# Low-Cost, Accurate, Effective Treatment of Hypertensive Cerebral Hemorrhage With Three-Dimensional Printing Technology

**DOI:** 10.3389/fneur.2021.608403

**Published:** 2021-02-25

**Authors:** Ke Li, Xiangqian Ding, Qingbo Wang, Gangxian Fan, Wei Guo, Chenglong Li, Meng Li, Zefu Li

**Affiliations:** ^1^Department of Neurosurgery, Binzhou Medical University Hospital, Binzhou, China; ^2^Department of Neurosurgery, First Affiliated Hospital of Kunming Medical University, Kunming, China; ^3^Department of Neurology, Binzhou Medical University Hospital, Binzhou, China

**Keywords:** hypertensive intracerebral hemorrhage, minimally invasive, three dimensional printing, individualization, accuracy

## Abstract

**Background:** Hypertensive intracerebral hemorrhage (HICH) is an acute, severe neurosurgical disease. Puncture drainage of the hematoma has gradually been accepted as a surgical treatment for HICH because of its minimally invasive nature. The precision of the puncture is extremely high because of particular physiological functions. This study was performed to explore the effect of a navigation mold created by three-dimensional printing (3DP) technology in the surgical treatment of HICH.

**Material and methods:** We conducted a retrospective analysis of all consecutive patients with ICH treated with minimally invasive surgery using 3DP navigation or craniotomy to remove the hematoma through a small bone window at the Binzhou Medical University Hospital from June 2017 to March 2019. In total, 61 patients were treated with minimally invasive surgery using 3DP navigation (3DP group), and 67 patients were treated with craniotomy to remove the hematoma through a small bone window (craniotomy group). A comparative study of the two groups was conducted to assess the preoperative and postoperative conditions.

**Results:** The duration of the surgery was significantly longer in the craniotomy group than in the 3DP group (3.27 ± 1.14 h vs. 1.52 ± 0.23 h). Postoperative complication rates were significantly lower in the 3DP group than in the craniotomy group (18.0 vs. 34.3%). Moreover, the rate of patients with a Glasgow Outcome Scale score ≥4 points was not statistically significantly different in the two groups.

**Conclusion:** Minimally invasive surgery assisted by 3DP navigation to treat patients with HICH appears to be safe and effective. The 3DP technique may improve the individualization and accuracy of the surgery.

## Introduction

Hypertensive intracerebral hemorrhage (HICH) is a common critical neurosurgical illness with high mortality and disability rates (1–3). At present, surgical treatment for HICH mainly involves removing the hematoma by craniotomy or minimally invasive surgery (4–6). Although craniotomy removal quickly relieves the compression of the hematoma on brain tissue and effectively stops bleeding, the operation is lengthy, which causes great trauma to the patient; it is also expensive (7). Minimally invasive surgery is widely performed as surgical treatment of HICH because it is a short procedure that causes little trauma and is low cost (8–10).

With the advances in medical technology in recent years, navigation and stereotactic, robotic, and other technologies have been introduced to the field of neurosurgery, which have greatly improved the accuracy of minimally invasive surgery and significantly benefitted patients. However, the role of this type of surgery has been limited because the equipment required is expensive and thus difficult to promote and popularize in developing countries.

Three-dimensional printing (3DP) technology has recently become popular in biomedical studies. It is a rapid, prototyping technology based on digital model files that can be used to print a solid object with complex geometry through layered processing. It has been gradually applied to the medical field because of its speed and accuracy (11–13). We have used 3DP technology to construct navigation molds that assist in intracranial hematoma puncture, hoping to achieve more individualized and precise treatment that retains the therapeutic effects of stereotactic, navigational, and other equipment.

## Materials and Methods

All patients provided written informed consent for the publication of all images and data, including any potentially identifiable images or data included in this article. The Medical Science Ethics Committee and Chinese Clinical Trial Registry approved this study (ChiCTR1800014543, 20/01/2018). The participating patients or their relatives or legal guardians also provided written informed consent for performance of the operation. All methods of this study were performed in accordance with the Guidelines for the Diagnosis and Treatment of Cerebral Hemorrhage in China in 2014.

### Patients

We conducted a retrospective analysis of all consecutive patients with ICH treated at the Binzhou Medical University Hospital from June 2017 to March 2019. The inclusion criteria were (1) a diagnosis of HICH based on the Guidelines for the Diagnosis and Treatment of Cerebral Hemorrhage in China formulated by the Chinese Medical Association in 2014 and confirmed by brain computed tomography (CT); (2) location of the bleeding site at the basal ganglia (i.e., the hematoma did not enter the ventricle); (3) absence of a cerebral hernia, cerebrovascular malformation aneurysm, or stroke; (4) first-onset lesion; and (5) treatment with minimally invasive surgery using 3DP navigation or craniotomy to remove the hematoma through a small bone window. In total, 61 patients were treated with minimally invasive surgery using 3DP navigation (3DP group), and 67 patients were treated with craniotomy to remove the hematoma through a small bone window (craniotomy group). A comparative study of the two groups was conducted to assess the preoperative and postoperative conditions.

### Minimally Invasive Surgery

#### Production of 3DP Guide Plate

**Figure 1:**
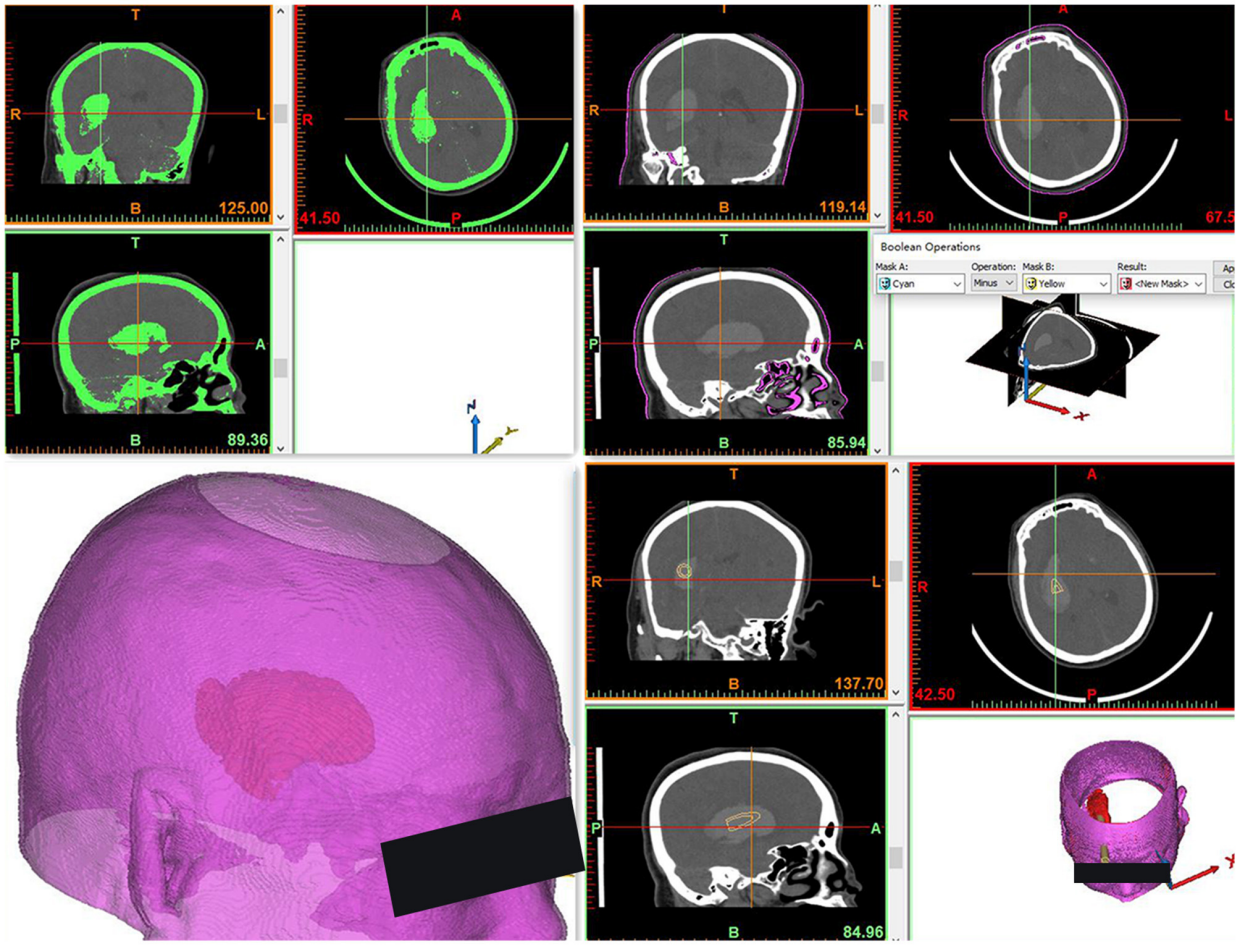
Three-dimensional reconstruction model of the hematoma and cranium, designed for optimal puncture passage. The end of the puncture passage points to the hematoma center and avoids the frontal sinus and important blood vessels.

**Figure 2:**
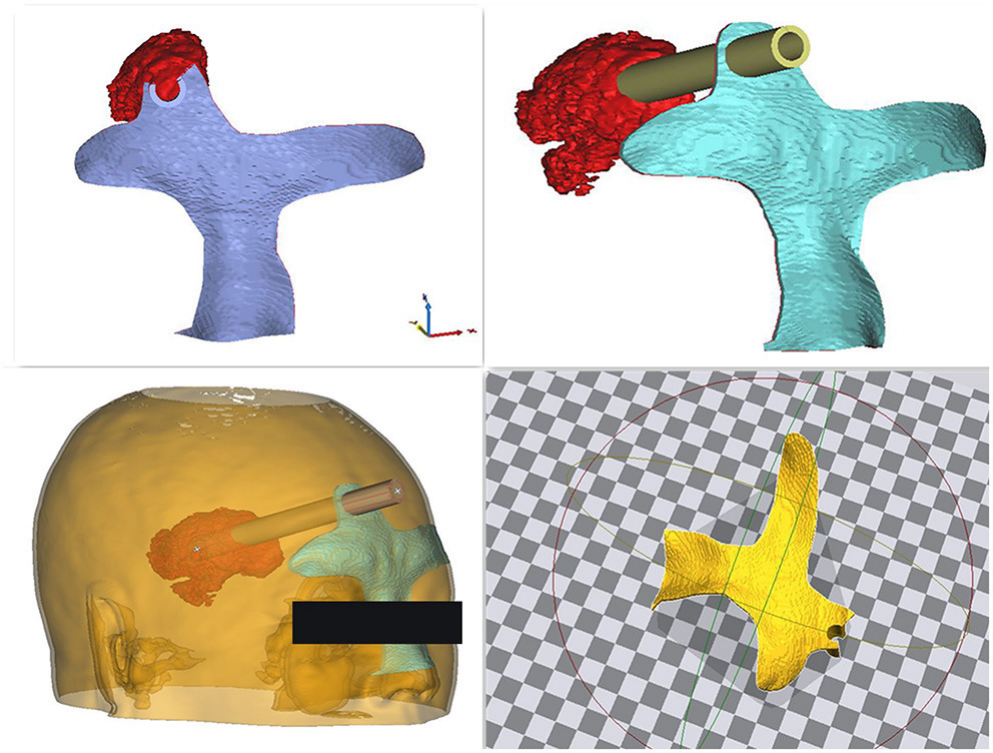
Construction of the navigation mold. The puncture guide plate was applied as closely as possible to the patient's face.

**Figure 3:**
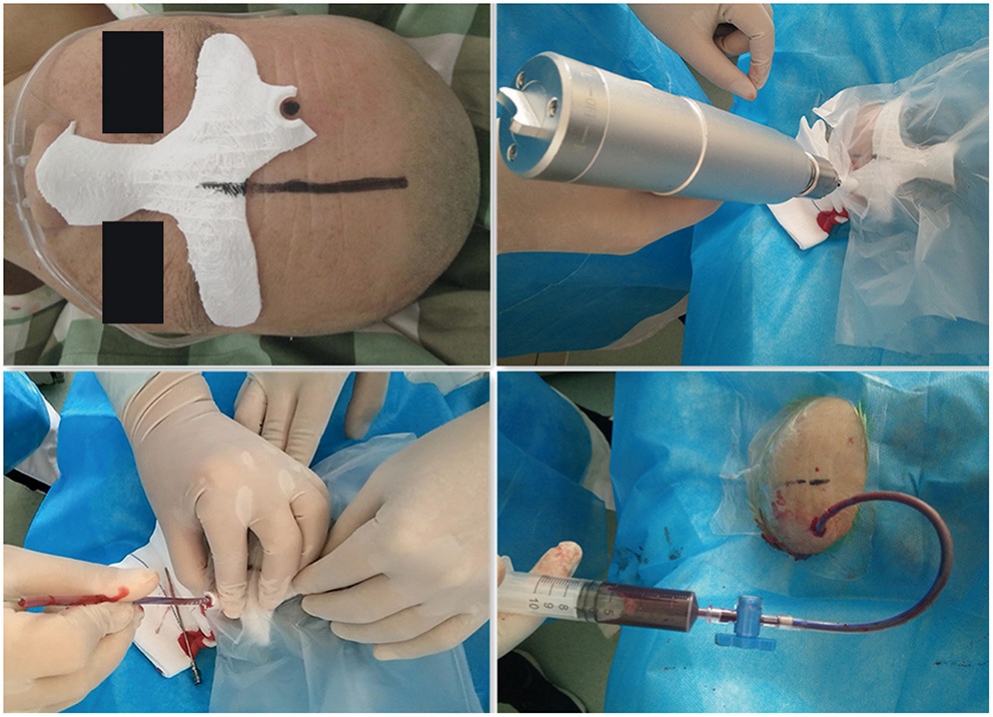
Surgical procedure for the three-dimensional printer (3DP) group.

To create the 3DP guide plate, Digital Imaging and Communications in Medicine format data obtained from CT scans were imported into Mimics 17.0 software embedded in the computer system to construct the skull, brain tissue, and hematoma in a 1:1 ratio *via* threshold selection (Figure 1). Bone markers such as the nasal root and bilateral eyebrow arches were reconstructed to fix on the patient's face (Figure 2). The puncture point was generally 3–5 cm from the eyebrow arch. The puncture channel avoided important structures, such as the frontal sinus. The guide plate for surgery was designed by a Boolean logic operation and then printed by the 3D printer (Figure 3).

#### Surgical Procedure

**Figure 4:**
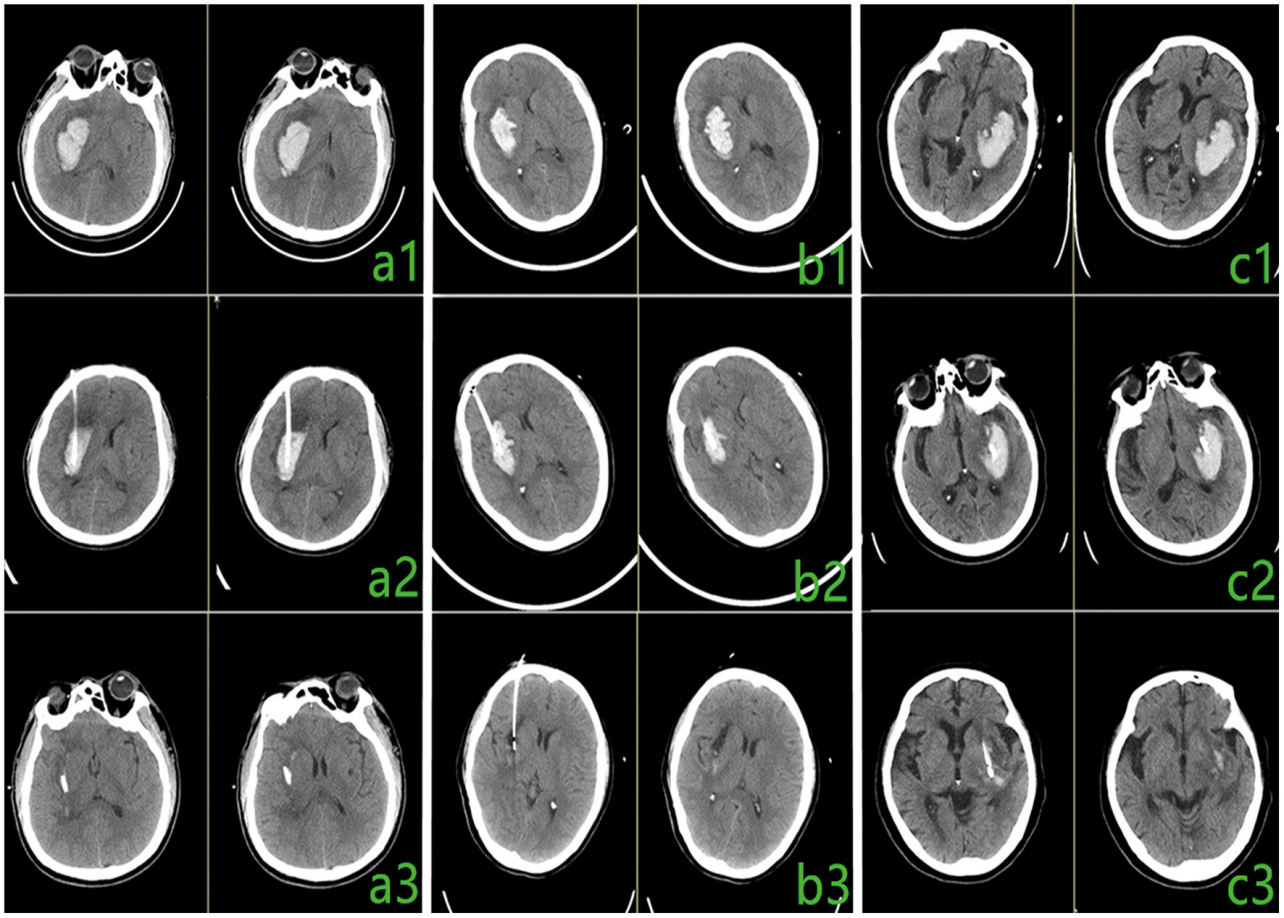
Comparison of preoperative and postoperative computed tomography (CT) results. (**a1**–**c1**) Preoperative CT. (**a2**–**c2**) Postoperative CT, 6 h after surgery. (**a3**–**c3**) Postoperative CT, 3 days after surgery.

The diagnosis of HICH was confirmed by preoperative brain CT (Figures 4a1–c1). After appropriately positioning the patient, we placed the 3D individualized model close to his or her face, ensuring that the bridge of the nose and zygomatic arch were accurately positioned. Next, along the puncture channel, we placed a marker at the patient's frontal puncture point. The surgical area was then routinely disinfected. Lidocaine 2% was used for local infiltration anesthesia. After confirmation of successful anesthesia, a 0.5-cm transverse incision was made at the center of the puncture point on the forehead. The model was then positioned on the patient's face and kept as close to the patient's skin as possible. A cranial drill was then introduced into a bone hole to penetrate the dura mater, moving in the direction of the puncture channel. A drainage tube was slowly inserted in the same direction until the desired depth was reached (guide-channel length plus intracranial length), and advancement of the tube was stopped when dark red liquid began to flow.

#### Adjuvant Therapy

Brain CT was performed 6 h postoperatively (Figures 4a2–c2). Urokinase was injected through the drainage tube for 2–3 days (once or twice a day) after the surgery. Strict sterility was observed during the operational procedures. Brain CT was performed to check the intracranial condition after the injection of urokinase (Figures 4a3–c3).

### Surgical Hematoma Removal *via* Small Bone Window

To remove the hematoma with conventional surgery, a 5-cm straight skin incision was made in the patient's temporal region. One or two bone holes were then drilled, and a milling cutter was used to create a bone window with a diameter of about 3 cm. A cortical ostomy was performed in the superior or middle temporal gyrus, and the hematoma was removed under microscopic guidance. A drainage tube was placed inside the hematoma, and the bone flap was repositioned.

### Evaluation of Surgical Methods

The safety and feasibility of minimally invasive surgery with 3DP individualized guidance was evaluated based on the efficiency and treatment outcomes (compared with those of craniotomy). The efficiency of the surgery was judged according to the duration of the surgery. We evaluated the treatment outcome based on the mortality rate, number of postoperative complications (i.e., pulmonary infections, rebleeding, and intracranial infection), Glasgow Outcome Scale (GOS) score at 30 days postoperatively, and duration of postoperative hospitalization.

Follow-up methods included telephone assessments and scheduled outpatient head CT examinations. Clinical efficacy evaluation referenced the GOS, which included the following: (1) death, (2) vegetative survival (i.e., minimal response, no sleep or wake cycle, if the eyes open on command), (3) severe disability (conscious but disabled and requiring assistance in daily life), (4) mild disability (able to live independently and work under protection), and (5) good recovery (a return to normal life despite minor defects).

### Statistical Analysis

IBM SPSS 21.0 statistical software (IBM Corp., Armonk, NY, USA) was used for statistical analysis of data, which are expressed as mean ± standard deviation. We performed Student's *t*-test to analyze the differences between the two groups. Count data were compared between the two groups using the χ^2^ test, with *P* < 0.05 indicating a statistically significant difference.

## Results

### Comparison of Preoperative Data

Among the 61 patients in the 3DP group (37 men, 24 women; mean age, 58.4 ± 9.5 years; range, 44–79 years), the mean systolic blood pressure was 174 ± 22 mmHg, the mean hematoma volume was 36.3 ± 6.6 ml, and the mean Glasgow Coma Scale (GCS) score was 12 ± 2 on admission. Among the 67 patients in the craniotomy group (42 men, 25 women; mean age, 55.5 ± 9.6 years; range, 38–73 years), the mean systolic blood pressure was 178 ± 24 mmHg, the mean hematoma volume was 36.7 ± 6.6 ml, and the mean GCS score was 11 ± 3 on admission. No significant differences in sex, age, systolic blood pressure, hematoma volume, or GCS score were observed between the two groups (Table 1).

**Table 1:** Comparison of preoperative data in two groups of patients.

**Factors**	**3DP group**	**Craniotomy group**	***t/χ*^**2**^ value**
	**(*n* = 61)**	**(*n* = 67)**	
Sex (male %)	37 (60.7)	42 (62.7)	*χ^2^* = 0.056
Age, years	58.4 ± 9.5	55.5 ± 9.6	*t* = 1.72
Systolic pressure, mmHg	174 ± 22	178 ± 24	*t* = −1.01
Hematoma volume, ml	36.3 ± 6.6	36.7 ± 6.6	*t* = −0.34
GCS score	12 ± 2	11 ± 3	*t* = 1.59

*There was no statistically significant difference between the two groups*.

### Comparison of Postoperative Data

The patients in the 3DP group underwent surgical drainage of their hematomas under local infiltration anesthesia. The puncture channel accurately located the position of the hematoma. All patients in the craniotomy group underwent complete surgical drainage under general intravenous anesthesia. Table 2 compares the mean operation time, mean postoperative hospitalization duration, complication rate, and proportion of patients with a GOS score of ≥4 points between the 3DP and craniotomy groups. The mean operation time, mean postoperative hospitalization duration, and complication rate were significantly different between the two groups (*P* < 0.05). However, the proportion of patients with a GOS score of ≥4 points was not significantly different between the two groups.

**Table 2:** Comparison of postoperative data in two groups of patients.

	**3DP group**	**Craniotomy group**	***t/χ*^**2**^ value**	***P*-value**
	**(*n* = 61)**	**(*n* = 67)**		
The preparation time of 3D model (3D modeling and printing), h	1.03 ± 0.09			
The total operation duration, h	1.52 ± 0.23	3.27 ± 1.14	*t* = −11.68	<0.001^*^
Postoperative hospitalization, days	20.54 ± 7.08	23.86 ± 10.72	*t* = −2.04	0.044^*^
Overall complication rate	11 (18.0%)	23 (34.3%)	*χ^2^* = 4.35	0.037^*^
Complication rate of pulmonary infection	8 (13.1%)	17 (25.4%)	*χ^2^* = 3.05	0.081
Complication rate of Intracranial infection	2 (3.3%)	3 (4.5%)		
Rebleeding rate	1 (1.6%)	3 (4.5%)		
Mortality rate	0	2 (3.3%)		
Rate of patients with a GOS ≥ 4 points	31 (50.8%)	38 (56.7%)	*χ^2^* = 0.45	0.503

*Compared with the control group*,

* *P < 0.05*.

## Discussion

HICH is a critical illness commonly seen by neurosurgeons. Early release of the compression of the hematoma on the brain tissue is a key factor in improving a patient's prognosis (14, 15). At present, surgical treatments for HICH mainly include removal of the hematoma *via* craniotomy through a small bone window, large-bone craniotomy, and drainage of the hematoma *via* minimally invasive puncture drainage. Large bone craniotomy is the traditional surgical procedure. Although this technique provides good visualization of the surgical field, it is not the preferred procedure because of its resultant trauma and postoperative complications (16).

With the continuing development of microsurgical techniques, removal of a hematoma *via* a craniotomy through small bone window has made significant progress and has offered obvious surgical benefits to patients. With the development of modern imaging technology and the introduction of stereotactic, navigation, robotic, and other procedures, minimally invasive surgery to accomplish puncture drainage is increasingly being applied in the clinical setting. However, this procedure shows no significant difference in surgical benefits compared with small bone window surgery (17).

Minimally invasive puncture takes only a short time, causes little trauma, and has a low postoperative complication rate and a short recovery time. However, because stereotactic, navigation, and robotic devices are expensive, promotion of their use in developing countries has been difficult. The accuracy of empirical puncture is not high and has become the main factor affecting patients' prognoses (16).

Three-dimensional printing technology has been increasingly applied in aerospace, medicine, automotive development, electronics, and other fields since the mid-1990's. Continuous improvements in 3DP technology have increased its clinical value because of the ability to deliver individualized, precise medicine. Thus, it is being widely applied clinically (18, 19). We introduced 3DP technology to the clinical diagnosis and treatment of neurosurgical patients and created a navigation mold for puncturing and draining hemorrhagic hematomas. This technique can improve the precision of puncture surgery and plays a role in the functions of stereotactic, navigation, robotic, and other equipment. The cost of a 3D printer is only about 700 USD, and the cost of printing a navigation mold is <100 RMB (14 USD). Because of the low cost of the 3DP equipment and the associated consumables, this technology is easy to promote in developing countries, thereby bringing precise treatment to most of these patients. Because our technique does not have strict posture requirements, it can be performed under local anesthesia, which reduces its cost. The average cost of surgery in the 3DP group was half that in the craniotomy group.

The present study revealed no significant difference in the prognosis of patients who underwent 3DP navigation mold-assisted puncture surgery vs. small bone window surgery to remove hematomas. The 3DP-guided surgery could be completed under local infiltration anesthesia to shorten the whole operation time and reduce the risks associated with anesthesia. Craniotomy must be performed under general anesthesia, which increase the operation time; additionally, the risk of general anesthesia is high. Because of the short operation time and minimal trauma in the 3DP group, the incidence of complications was significantly lower, and the mean hospitalization time was significantly shorter than those in the craniotomy group.

## Conclusion

Using individualized 3DP guidance, minimally invasive surgery to treat patients with HICH appears to be technically feasible and effective. This procedure is more advantageous than craniotomy because of its minimal invasiveness, superior efficacy, low anesthesia risk, and low complication rate. It is more accurate than normal minimally invasive surgery without navigation, which reduces the number of punctures and trauma of most puncture wounds. The 3DP technique may improve the individualization and accuracy of minimally invasive surgery.

## Data Availability Statement

The original contributions presented in the study are included in the article/supplementary material, further inquiries can be directed to the corresponding author/s.

## Ethics Statement

The studies involving human participants were reviewed and approved by the ethics committee of Binzhou Medical University Hospital. The patients/participants provided their written informed consent to participate in this study. Written informed consent was obtained from the individuals for the publication of any potentially identifiable images or data included in this article.

## Author Contributions

QW and XD drafted the manuscript. QW, GF, WG, CL, and ML completed the operation. XD, GF, and WG performed the data collection and data analysis. KL is responsible for the revision and data processing of the article. ZL participated in the design of this study and helped to check the manuscript. All authors read and approved the final manuscript.

## Conflict of Interest

The authors declare that the research was conducted in the absence of any commercial or financial relationships that could be construed as a potential conflict of interest.

## Abbreviations

3DP: three-dimensional printing
CT: computed tomography
GCS: Glasgow Coma Scale
GOS: Glasgow Outcome Scale
HICH: hypertensive intracerebral hemorrhage.
